# LncRNAs as Regulators of Autophagy and Drug Resistance in Colorectal Cancer

**DOI:** 10.3389/fonc.2019.01008

**Published:** 2019-10-02

**Authors:** Mercedes Bermúdez, Maribel Aguilar-Medina, Erik Lizárraga-Verdugo, Mariana Avendaño-Félix, Erika Silva-Benítez, Cesar López-Camarillo, Rosalío Ramos-Payán

**Affiliations:** ^1^Facultad de Ciencias Químico Biológicas, Universidad Autónoma de Sinaloa, Culiacán, Mexico; ^2^Facultad de Odontología, Universidad Autónoma de Sinaloa, Culiacán, Mexico; ^3^Posgrado en Ciencias Genómicas, Universidad Autónoma de la Ciudad de México, Mexico City, Mexico

**Keywords:** lncRNA, colorectal cancer, autophagy, chemoresistance, drug resistance, macroautophagy

## Abstract

Colorectal cancer (CRC) is a common malignancy with 1. 8 million cases in 2018. Autophagy helps to maintain an adequate cancer microenvironment in order to provide nutritional supplement under adverse conditions such as starvation and hypoxia. Additionally, most of the cases of CRC are unresponsive to chemotherapy, representing a significant challenge for cancer therapy. Recently, autophagy induced by therapy has been shown as a unique mechanism of resistance to anticancer drugs. In this regard, long non-coding RNAs (lncRNAs) analysis are important for cancer detection, progression, diagnosis, therapy response, and prognostic values. With increasing development of quantitative detection techniques, lncRNAs derived from patients' non-invasive samples (i.e., blood, stools, and urine) has become into a novel approach in precision oncology. Tumorspecific GAS5, HOTAIR, H19, and MALAT are novels CRC related lncRNAs detected in patients. Nonetheless, the effect and mechanism of lncRNAs in cancer autophagy and chemoresistance have not been extensively characterized. Chemoresistance and autophagy are relevant for cancer treatment and lncRNAs play a pivotal role in resistance acquisition for several drugs. LncRNAs such as HAGLROS, KCNQ1OT1, and H19 are examples of lncRNAs related to chemoresistance leaded by autophagy. Finally, clinical implications of lncRNAs in CRC are relevant, since they have been associated with tumor differentiation, tumor size, histological grade, histological types, Dukes staging, degree of differentiation, lymph node metastasis, distant metastasis, recurrent free survival, and overall survival (OS).

## Introduction

Cancer is one of the most deathly illness worldwide with an estimated 9.6 million deaths in 2018 (1). One of the most common is colorectal cancer (CRC) with 1.8 million cases and 862,000 deaths only during the last year (1). Development of CRC involves different genetic and epigenetic changes. Most cases are sporadic and show a slow development through the time, advancing from adenoma to carcinoma (2). Even though there are important progress in treatment and molecular mechanisms involved in CRC, the OS rate still remains relatively low (3, 4).

Chemotherapy has been widely used for cancer treatment, for instance, the fluoropyrimidine 5-fluorouracil (5-FU) is a first selection anticancer drugs for CRC treatment (5). Besides, new drugs such as cetuximab and panitumumab have been incorporated into clinical practice (6). Nevertheless, drug resistance acquisition is one of the main issues in effective chemotherapy (7). This due to different factors as Pharmacokinetic Resistance, that includes since absorption until, distribution, metabolism, and the excretion of drugs. In addition, the evolutionary resistance, a process that occurs in the tumor where the cells acquire the ability to survive chemotherapy, this through expression of different proteins, such as P-glycoprotein 1 (P-gp) also known as, multidrug resistance protein 1 (MDR1). Besides the physics of the tumor site is involved in chemotherapy resistance such as, number and morphology of vessels and blood viscosity, are important factors involved (8). Drug ineffectiveness could be the result from tumor-host interactions and a clear understanding of such an interaction will open new opportunities not only for the discovery of new drugs but also for new therapeutic strategies to overcome the development and evolution of resistance to cancer chemotherapy.

Autophagy is an important cellular response to stress or starvation and starts when organelles and proteins are sequestered in vesicles and delivered to lysosomes for degradation (9). New research revealed that autophagy has different functions in the development, maintenance, and tumor progression (10) and recently, autophagy induced by therapy has been shown as a new mechanism of resistance to chemotherapeutic drugs (11). Through carcinogenic process of CRC, autophagy could promote tumor survival or cancer cell death, and it depends on the tumor type, stage, and the metabolic setting (12).

Non-coding RNAs (ncRNAs) represent 99% of total transcribed RNAs in the human genome, being the principal components of the human transcriptome (13). Recently, ncRNAs have shown to play key roles in important biological processes by interfering with gene expression in several cancer types (14, 15).

The best characterized of the “expanding universe” of ncRNAs are the ~22 nucleotide microRNAs (miRNAs) and the long non-coding RNAs (lncRNAs). The lncRNAs are classified as >200 nucleotides in length and are involved in a wide variety of molecular genetics and cellular processes in many aspects of gene regulation, including imprinting, epigenetic modulation, transcription, mRNA splicing, and tracking between the nucleus and cytoplasm (15–18). Moreover, lncRNAs are involved in variety biological processes such as, proliferation, differentiation, apoptosis, invasion, and metastasis.

Recently, lncRNAs have been implicated in tumor-drug resistance and autophagy in different types of cancer including CRC (16, 19–22). Therefore, the aim of this review is to compile the current knowledge about lncRNAs and their implication on chemoresistance and autophagy in CRC. To this end, we searched on PubMed, PMC, Web of Science, Google scholar, and EMBASE up to July 2019 for pertinent articles using the keywords as follows: (lncRNA or long non-coding RNA) and (CRC or colorectal cancer) and (autophagy or autophagia) and (chemoresistance or drug resistance). The titles and abstracts were screened, and we acquired the relevant full-text manuscripts for perusal.

## Long Non-coding RNAs

### Biogenesis, Classification, and Function

LncRNAs include different types of RNA polymerase II (Pol II)-transcribed molecules with sizes over 200 nt in length. It has been reported an estimated abundance of 5,400 to more than 10,000 lncRNAs transcripts in humans (23, 24). All mammalian lncRNAs share a few structural, functional, or mechanistic characteristics among them. They often harbor a poly-A tail and can be spliced, similar to mRNAs (25). Besides, they regulate gene expression at transcriptional and post-transcriptional levels in multiple biological processes and cellular contexts (26–28).

**Figure 1:**
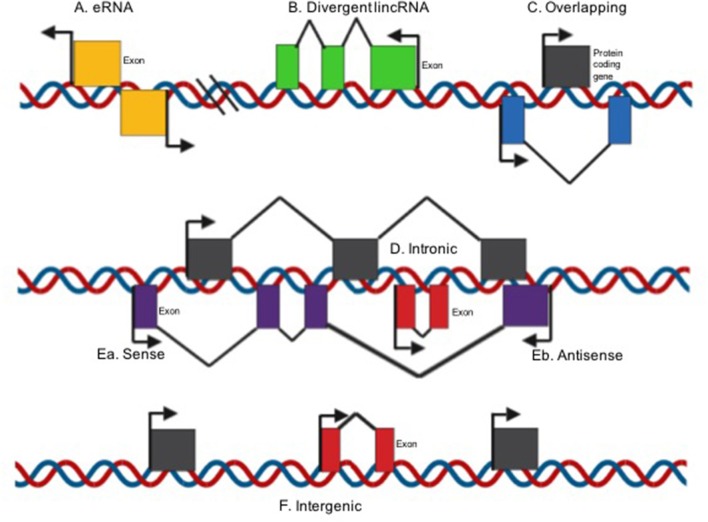
LncRNA classification on their structural origin context [modified image from Spurlock et al. (29)]. **(A)** Enhancer RNAs can be transcribed in one or two senses 1D-eRNAs and 2D-eRNAs, respectively, at genomic transcriptional enhancers, frequently in close proximity to protein-coding genes; **(B)** Divergent when the lincRNA and nearby protein coding gene are transcribed on opposite strands; **(C)** overlapping when a protein-coding genes is included in the intron of a lncRNA; **(D)** Intronic when the whole sequence of the lncRNA belongs to the intron of a protein-coding gene; Ea. Sense or **(E)** antisense if the lncRNA is located between one or more exons of another transcript on the same sense or antisense strand; **(F)** intergenic when a lncRNA sequence belongs to two genes as a distinct unit.

Spurlock et al., classified LncRNAs based on their structural origin context (Figure 1). Overlapping when a protein-coding genes is included in the intron of a lncRNA (29, 30), divergent when the lncRNA and neighboring protein coding gene are transcribed on opposite strands (31), intronic when the whole sequence of the lncRNA belongs to the intron of a protein-coding gene (32), intergenic when a lncRNA sequence belongs to two genes as a distinct unit (33), and sense (34) or antisense (35) when the lncRNA is located between one or more exons of another transcript on the same sense or antisense strand (36–38). Lastly, enhancer RNAs can be transcribed in one or two senses, 1D-eRNAs and 2D-eRNAs, respectively, at genomic transcriptional enhancers, frequently very close to protein-coding genes (39).

**Figure 2:**
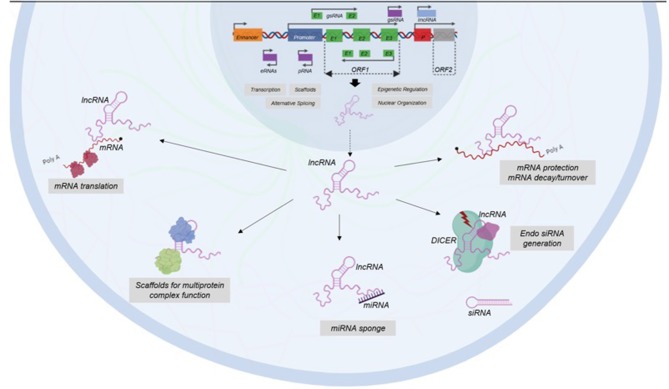
Classification of LncRNAs based on their functions. LncRNAs participate in transcription, epigenetic regulation, nuclear organization, and alternative splicing at nuclear level. In cytoplasm, LncRNAs have functions as enhancers of mRNA translation, scaffolds of protein complex, miRNA sponges, generators of endo siRNA, and protectors of mRNA.

It has been shown that lncRNAs functions depend on their subcellular location (26). There is evidence in human cell lines using single molecule RNA fluorescence *in situ*-hybridization that revealed a wide range of subcellular localization patterns, including nucleus, cytoplasm and both (40). Nevertheless, it is most common to catalog lncRNAs based on similar action mechanisms (25) (Figure 2).

Some lncRNAs have a very important role in nuclear structure, since they help to the structure of nuclear speckles, paraspeckles, and interchromatin granules (41). Another nuclear lncRNAs are able to regulate gene expression by epigenetic mechanisms and recruiting chromatin-modification factors in order to switch-on or switch-off different loci (42). Besides, there are other types of stable lncRNAs, such as competing endogenous RNAs (ceRNAs) and circular RNAs, which are accumulated in the cell acting as decoys or sponges for miRNAs modulating gene expression (43).

LncRNAs also has an important role in transcription since they help in assembling transcriptional activators and repressors for modulating the activation of transcription (44). Besides, lncRNAs are able to modulate gene expression post-transcriptionally by interfering with RNA-binding proteins to impact splicing and translation and by modulating the translation and stability of partially complementary mRNAs (45, 46). In addition, some lncRNAs function post-transductionally in order to regulate protein turnover to enhance ubiquitination (47).

### Detection Methods

The lncRNAs importance in cancer characteristics such as progression, autophagy, and chemotherapy resistance has been established thanks to more advanced detection technologies. The main two methods for lncRNAs detection are microarrays and RNA sequencing (RNA-seq), Microarrays contain probe sequences that match with lncRNAs (48). Whereas, RNA-seq provides comprehensive coverage of whole transcriptomes compared to microarrays. Due to unbiased genome-wide screening, it is possible to exclude ribosomal RNAs (rRNAs) from total RNA to enhance it, including protein-coding genes and lncRNAs. Besides, it is possible to enrich mRNAs using oligo-dT beads with poly A tails, giving as a result the detection of protein-coding genes and lncRNAs with poly A tails that are nearly 60% of total lncRNAs (49, 50).

Since lncRNAs has been described as biomarkers in several types of cancer, non-invasive detection methods have been developed (Table 1) for early diagnostic, evolution, and poor prognosis of cancer (62). Thus, there are several carcinomas that can be detected by specific serum circulating lncRNAs (Table 1) (63). Moreover, lncRNAs are detectable in urine and may serve as biomarker predictor in T-cell mediated kidney transplant rejection as well as bladder cancer tumor-stage (64, 65). In addition, US Food and Drug administration (FDA) has recently approved PCA3 lncRNA as a biomarker for prostate cancer in urine (66, 67) showing better sensitivity and specificity than Prostate-specific antigen (PSA) blood test (68). Whole saliva also represents a source for cancer biomarkers by lncRNAs detection, given this, saliva contains certain lncRNAs that can be used as biomarkers for oral squamous cell carcinoma diagnosis such as HOTAIR, which presence in saliva samples is correlated with high levels in metastatic tissues (69).

**Table 1:** Circulating lncRNAs detected in serum in different types of cancer.

**LncRNA**	**Associated cancer**	**References**
RP11-04K16.1, LOC_012542, PVT1	Cervical cancer	(51, 52)
SNHG1, RMRP	Lung	(53)
H19	Multiple myeloma	(54)
PCA3, BCAR4, CRNDE-h, LNCV6_116109, LNCV6_98390, LNCV6_38772, LNCV_108266, LNCV6_84003, LNCV6_98602, u50535	Colorectal	(55–58)
H19, lncUEGC1	Gastric	(59, 60)
LINC00161	Hepatocellular carcinoma	(61)

## LNCRNAs in Colorectal Cancer

LncRNAs play key roles regulating gene expression during cell development and differentiation, regulating or maintaining cellular homeostasis (70, 71). Abnormal expression of lncRNAs has been reported in numerous cancer types such as; hematopoietic, urologic, lung, liver, breast, ovarian, and colorectal (72–79). Alterations of these molecules are studied in CRC in order to obtain clinical biomarkers for diagnostic, prognostic, and therapeutic applications (80, 81). Multiple lncRNAs have been related with CRC as important clinical and mechanistic molecules (Table 2) and there are some lncRNA that are strongly associated to CRC and presented below.

**Table 2:** Important lncRNAs involved in CRC.

**lncRNA**	**Status of expression**	**Participation in CRC**	**References**
XLOC_010588	Upregulated	Associated with metastasis, poor prognosis, invasion, migration, and the progression of CRC via EMT pathway	(82)
FTX	Upregulated	Tumor diameter, TNM stage, the lymph node, and distant metastasis and poor prognosis of patients with CRC. *In vitro*, promotes CRC cell proliferation, migration, invasion, and interacts with miR-215 and vimentin	(83)
BLACAT1	Upregulated	Proliferation, both *in vitro* and *in vivo*, and have a role in G1/G0 arrest by binding to EZH2	(84)
lnc-CRCMSL	Downregulated	Overexpression restricts tumor growth and metastasis *in vivo* and *in vitro* and the silencing accelerates CRC cell proliferation and migration. Also, mediates suppression EMT process by HMGB2	(85)
DANCR	Upregulated	Promotes proliferation and metastasis in CRC. DANCR promotes HSP27 expression and its mediation of proliferation/metastasis via miR-577 sponging. *In vivo*, DANCR promotes CRC tumor growth and liver metastasis	(86)
lnc-DILC	Upregulated	Inhibits the growth and metastasis of CRC cells. Knockdown, facilitates the proliferation and metastasis of CRC cells. Lnc-DILC is a CRC suppressor by inactivating IL-6/STAT3 signaling	(87)
kcna3	Downregulated	Higher TNM grade and the higher occurrence rate of lymphatic metastasis and distant metastasis, and shorter OS. Overexpression, inhibits proliferation, migration and invasion and induces cell apoptosis *in vitro*, and represses CRC tumor growth *in vivo*. Also, exerts a tumor-inhibit role in CRC progression through down-regulating YAP1 expression	(88)
Loc554202	Downregulated	Associated with advanced TNM and a larger tumor size. The overexpression decreases the cell proliferation and induces apoptosis *in vitro* and delay tumorigenesis *in vivo*. Regulates cell apoptosis through the activation of specific caspase cleavage cascades	(89)
MAPKAPK5- AS1	Upregulated	Greater tumor size and advanced TNM in CRC patients. Knockdown, inhibits proliferation and causes apoptosis in CRC cells. Also, p21 is a target of MAPKAPK5- AS1	(90)
ZNFX1-AS1	Upregulated	Associated with aggressive tumor phenotype and poor prognosis in CRC. Knockdown inhibits cell proliferation and invasion *in vitro*, and tumorigenesis and metastasis *in vivo*. ZNFX1-AS1 works as a ceRNA for miR-144, inhibiting to EZH2	(91)
u50535	Upregulated	Activates CCL20 signaling to promote cell proliferation and migration in CRC	(58)
DUXAP10	Upregulated	Positively correlated with advanced pathological stages, larger tumor sizes, and lymph node metastasis. Knockdown inhibits cell proliferation, induces cell apoptosis and increase G0/G1 cells. DUXAP10 silencing inhibits tumor growth *in vivo*, also promotes CRC cell growth and reduces cell apoptosis through silencing the expression of p21 and PTEN by binding LSD1	(92)
NNT-AS1	Upregulated	Correlated with lymph node metastasis, TNM stage, vessel invasion and differentiation, Also, is an independent predictor of OS and progression free survival. Knockdown, inhibits CRC cell proliferation, migration and invasion *in vitro* and suppress tumor growth and metastasis in nude mice by NNT-AS1-mediated activating of MAPK/Erk signaling pathway and EMT	(93)
91H	Upregulated	Associated with distant metastasis and poor prognosis in patients with CRC. Also, is an independent prognostic indicator and of distant metastasis. *In vitro*, knockdown of 91H inhibits the proliferation, migration, and invasiveness of CRC cells	(94)

In this regard, the growth arrest-specific transcript 5 (GAS5), is located at 1q25, with a length of 630 nt (95). GAS5 is upregulated during growth arrest induced by the absence of growth factors or serum starvation. It has been shown that GAS5 binds to the DNA-binding domain of the glucocorticoid receptor (GR) and acts as a decoy glucocorticoid response element (GRE), therefore it can compete with DNA GREs for binding to the GR (95). This lncRNA is able to inhibit cell proliferation and promote apoptosis, by acting as tumor suppressor (96). Nowadays, researches demonstrate that GAS5 is downregulated in several cancer cells such as, breast cancer, prostate cancer, and renal carcinoma (97–99).

In human CRC tumor tissues, Gas5 has been found downregulated and it is correlated with tumor size, TNM staging, lymph node metastasis, low histological grade and less OS (100–104). Besides, overexpression of GAS5 shows that could inhibit cell proliferation *in vitro* and *in vivo* (102), prevent migration and invasion (100, 105), and promotes apoptosis (100, 101, 103) through inhibition of mRNA expression of Akt and Erk and protein expression of p-Akt and p-Erk, giving as a result A pho-Casp9 protein expression and inhibition of pho-Casp3 protein expression (100). Another mechanism of GAS5 to inhibit the apoptosis could be through the GAS5/miR-182-5p/FOXO3a axis, since GAS5 acts as ceRNA of miR-18-5p, which regulates a pro-apoptotic transcription factor named FOXO3a, and target directly the PI3K-AKT signaling pathway (101).

In the case of HOTAIR (Homeobox Transcript Antisense Intergenic RNA), a 2.2 kb lncRNA, is transcribed from the mammalian HOXC gene cluster located in 12q13.13 (106). It participates in epigenetic regulation of gene transcription and interacts on its 5′ end with Polycomb repressive complex 2 in order to remodel chromatin and guarantee silencing of HOX genes during embryonic development. On 3′ end HOTAIR interacts with histone demethylase (107). Evidence shows that HOTAIR exhibits an oncogenic role in renal, breast, gastric, lung, and ovarian cancer (108–112).

HOTAIR is overexpressed at high levels in CRC (113–116) and some studies show that HOTAIR is only overexpressed in right (proximal) CRCs samples (117). This overregulation has been associated to lymph node and tumor node metastasis, distant metastases, Duke's staging, histological types, the degree of differentiation (113), and unfavorable prognosis (114, 118). *In vitro*, the inhibition of its expression shows decreased proliferation, invasion, and migration, as well as low cyclin E and CDK2 expression, increased apoptosis and p21 expression (113). Besides, HOTAIR promotes tumorigenesis and aggressiveness (114). This lncRNA directly harbors miR-326 binding sites and regulates FUT6 expression, a specific fucosyl transferase. The HOTAIR/miR-326/FUT6 axis modifies α1, 3-fucosylation of CD44, which triggers PI3K/AKT/mTOR pathway mediating CRC malignancy (114). In addition, HOTAIR knockdown and miR-203a-3p upregulation in CRC cell lines produces inhibited Wnt/β-catenin signaling, cell proliferation, and reduced chemoresistance (116).

The H19 gene is located on 11p15 and plays pivotal roles in embryonal development and growth regulation (119, 120). The H19 gene encodes for a processed 2.7 kb RNA (121). H19 is highly expressed from the onset of embryogenesis to fetal life in vital organs such as the fetal adrenal, liver, and placenta but is downregulated postnatal stages (122). Recent evidence shows that H19 is upregulated in several cancers as, esophageal cancer, hepatocellular carcinoma, ovarian cancer, bladder cancer, and breast cancer (123–127).

It has been demonstrated that H19 is upregulated in CRC tissues compared with adjacent noncancerous tissues (9, 128, 129). Data from The Cancer Genome Atlas (TCGA) shows that H19 is the lncRNA with the most substantial correlation to CRC patient survival (130), serving as an independent predictor for OS and disease-free survival (DFS) (9, 131). Besides, this lncRNA has been related with poor prognosis (132).

Besides, miR-200a binds H19 and inhibits its expression, thus decreasing proliferation of CRC cells, also H19 regulates the expression and activity of β-catenin by competitive binding to miR-200a (128). In addition, depletion of H19 inhibits cell viability and induces growth arrest whereas overexpression of H19 upregulates a series of cell-cycle genes. Moreover, H19 binds to eIF4A3 resulting in an abnormal cell-cycle-regulatory genes expression (131).

H19 promotes invasion and metastasis in CRC through activation of RAS-MAPK signaling pathway (133) and its overexpression in MTX-resistant colorectal cell line HT-29 prove that is involved in Metrotexate (MTX) resistance via activating Wnt/β-catenin signaling (134). The overexpression of H19 and miR-675 in CRC implies that both are important factors in the tumorigenesis of CRC since H19-derived miR-675, targets tumor suppressor RB (129).

Interestingly, mesenchymal-like cancer cells and primary CRC tissues show high expression of H19, whereas its stable expression accelerates tumor growth and enhances epithelial–mesenchymal transition (EMT) progression. Finally, H19 can function as ceRNA by antagonizing the functions of miR-138 and miR-200a, giving as a result the de-repression of Vimentin, ZEB1, and ZEB2 (135).

Finally, metastasis-associated lung adenocarcinoma transcript 1 (MALAT-1), is on 11q13 and transcribed from the nuclear-enriched transcript 2 (NEAT2), which has been identified as a prognostic factor in patients with stage I lung cancer (136, 137). It has been reported that this lncRNA is expressed in mouse and normal human tissues (137, 138) and its overexpression have been demonstrated in many cancer types including lung, cervical, liver, bladder and sarcomas of uterus (139–144), and correlated to metastasis (137).

The MALAT1 levels are up-regulated in human primary CRC tissues (136), being 2.26 times higher than noncancerous tissues (145), serving as a negative prognostic marker in stage II/III CRC patients, since, these patients show a high hazard ratio (HR) for OS and DFS (145). Moreover, upregulation of MALAT1 has been found in CRC tissues with lymph node metastasis (136). *In vitro*, MALAT1 could promote CRC cell proliferation, invasion, and migration through up-regulating SOX9 and down-regulating miR-145. On the other hand, cell cycle and apoptosis can be suppressed by MALAT1/miR-145/SOX9 axis (146). Furthermore, MALAT1 regulates proliferation, migration, and promotes tumor growth and metastasis in nude mice (136), this regulation could be through SFPQ and AKAP-9 as MALAT1 interact with SFPQ, hence releasing PTBP2 from the SFPQ/PTBP2 complex, facilitating cell proliferation and migration (147). AKAP-9 is overexpressed in CRC cells with metastatic potential and human primary CRC tissues with lymph node metastasis, and its knockdown blocks CRC cell proliferation, migration, and invasion mediated by MALAT1 (136).

Angiogenesis and the EMT to promote metastasis in CRC are enhanced by YAP1-induced MALAT1-miR126-5p axis since YAP1 forms a complex with β-catenin/TCF4 bound to the MALAT1 promoter, which can act as a sponge of miR-126-5p to induce SLUG, VEGFA, and TWIST expression (148). miR-20b-5p-mimic and si-MALAT1 give as a result attenuated microsphere formation and self-renewal capability, reduces the proportion of CSCs, downregulating the expression of stemness markers as Oct4, Nanog, Sox2, and Notch1, and cellular metabolism such as GLUT1, LDHB, HK2, and PKM2 in HCT-116 cells *in vitro*. Additionally, the administration of either si-MALAT1 or miR-20b-5p-mimic in a xenograft model based on BALB/c mice demonstrated that they can suppress tumorigenicity of HCT-116 cells *in vivo* (149).

As we reviewed above, HOTAIR, H19, and MALAT are overexpressed in CRC samples. Interestingly, HOTAIR and MALAT level expression are related to lymph node and tumor node metastasis (113, 136). In addition, H19 is considered as an important independent predictor for OS and DFS (9, 131), besides, H19 is the most significant lncRNA associated to CRC (130). Moreover, MALAT1 is one important negative prognostic marker in II/III CRC patients (145). Conversely, down regulation of Gas5 has been found in CRC and is associated with poor prognosis (100–104).

Interestingly, LncRNAs regulate multiples pathways in CRC as PI3K-AKT signaling pathway, that is regulated by GAS5, promoting apoptosis via GAS5/miR-182-5p/FOXO3a axis (101), as well as, PI3K/AKT/mTOR that is managed through HOTAIR/miR-326/FUT6 axis stimulating CRC (114). In addition, H19 regulates RAS-MAPK and Wnt/β-catenin signaling pathways, activating invasion, metastasis, and chemoresistance mechanism (133, 134). Another important axis is MALAT1/miR-145/SOX9 that mediates cell cycle and apoptosis (146).

## LNCRNA as Regulators of Autophagy in CRC

Autophagy is a basal physiological mechanism in normal cells that assure cellular homeostasis. Besides, autophagy is a very well-conserved catabolic process where the cell is self-digested through the removal of proteins or dysfunctional organelles (150). This process can also be, under specific circumstances (hypoxia, stress, and nutrient deprivation), a survival mechanism in which the cell recycles nutrients and energy (151).

There are three forms of autophagy based on its morphology, macroautophagy in which autophagosomes engulf cytoplasmic components and interact whit lysosomes for degradation, microautophagy in which there is a direct lysosomal membrane invagination to engulf damaged proteins, and chaperone-mediated autophagy which involves the translocation of soluble cytosolic proteins by chaperone-dependent selection across the lysosomal membrane (152–154).

LncRNAs generally modulate autophagy by regulating the expression of ATG genes which are important effectors in autophagy process (155, 156). Frequently, LncRNAs behaves as competing endogenous RNAs (ceRNAs) for modulating autophagy-related microRNAs (miRNAs). LncRNAs have a very important implication in autophagy regulation (155). For instance, activation of autophagy can be given by NBR2 via AMPK activation (157) or by repression of PI3K/AKT/mTOR pathway leaded by Ad5-AlncRNA, and PTENP1, whereas MEG3 and H19 enhances the opposite effect. Another LncRNAs involved in activation of autophagy are HOTAIRM1, PTENP1, and MALAT1, which increase the expression of ULK (158–162). Conversely, RISA suppress autophagy initiation through ULK1 inhibition (163). Additionally, key genes in autophagy such as ATG and adaptor proteins involved in later steps of autophagy regulation are affected by H19, MEG3, AK156230, PTENP1, and MALAT1(141, 158, 161, 164, 165).

It is clear that LncRNAs are non-canonical regulators and participates in keeping homeostasis in a variety of pathophysiological processes, but also they can be illness effectors, since they can interact directly with DNA, RNA, and proteins. In this regard, it has been demonstrated that autophagiaparticipates in cancer progression and drug resistance mechanisms (166). Besides, autophagy may suppress tumors (167), but also, their induction promotes tumorigenesis since it provide survival capacity of tumor under adverse microenvironment (168, 169).

In CRC, little is known about lncRNAs involved in autophagy, for instance, POU3F3, a lincRNA, is overexpressed in CRC tissue samples and when is silenced, autophagy is enhanced, suggesting the involvement of autophagy in the induction of apoptosis (170). Another lncRNA highly expressed in CRC is HAGLROS, which is correlated with shorter survival time of CRC patients and its decreased expression can produce apoptosis and suppress autophagy in CRC HCT116 cells by regulation of miR-100/ATG5 axis and PI3K/AKT/mTOR pathway (171).

UCA1 is also abnormally overexpressed in SW620 and HT29 CRC cell lines when compared to CCD-18Co. There is evidence that UCA1 downregulation inhibits the growth, apoptosis, and autophagy of CRC cell lines *in vitro*. Besides, UCA1 directly interacts with miR-185-5p downregulates its expression. Additionally, UCA1 could reverse this effect of miR-185-5p on the growth and autophagy, suggesting its involvement in the derepression of WISP2 expression and the stimulation of the WISP2/β-catenin signaling pathway (172).

Another lncRNA involved in CRC is KCNQ1OT1 (173), which is also upregulated. It has been demonstrated that expression patterns of Atg4B, which cleavages LC3 (thus promotes the formation of autophagosome) (174) is downregulated in CRC HCT116 and SW480 cells in KCNQ1OT1 knockdown cells. Besides, these cells treated with oxaliplatin, decrease cell viability, meaning that KCNQ1OT1 induce protective autophagy and chemoresistance. Finally, overexpression of KCNQ1OT1 is correlated with poor OS of CRC patients, suggesting that higher levels in patients make them resistant to chemotherapy treatments (173).

H19 is another upregulated lncRNA in CRC samples and has been correlated with patient OS suggesting that can predicts 5-FU chemoresistance. These findings reveal that SIRT1 (which is modulated by H19/miR-194-5p axis) dependent autophagy pathway can affect 5-FU resistance in CRC cells (9).

There is no doubt that LncRNAs are key molecules involved in regulation of autophagy in CRC. Nevertheless, more research in this field is needed to clarify interactions on regulation axis in order to understand complex processes in which autophagy is implicated, such as apoptosis and chemoresistance.

## LNCRNA as Regulator of Drug Resistance in CRC

Malignant CRC tumors develop pharmacological resistance, which is a complex phenomenon that triggers increase in DNA repair and loss of apoptosis induction, resulting from several factors that include individual variation in patients such as genetic and/or epigenetic differences within the tumors (7, 175, 176). Drug resistance is influenced by abnormal expression or mutation on efflux proteins, which reduce uptakes of drugs (177).

Chemotherapy for CRC depends on the stage of cancer; however, other factors are important as well. For stage 0 to II, surgical treatment alone might be successful, nonetheless, for stage II some oncologists opt for including 5-FU and leucovorin, oxaliplatin, or capecitabine if chemotherapy is needed (178–180). Treatment for stages III and IV includes chemo and/or targeted drugs, commonly include CAPEOX (capecitabine plus oxaliplatin), FOLFOX (oxaliplatin, 5-FU, and leucovorin), 5-FU and leucovorin, or capecitabine for stage III and FOLFIRI (leucovorin, 5-FU, and irinotecan), FOLFOXIRI (leucovorin, 5-FU, oxaliplatin, and irinotecan) plus some target drugs such as bevacizumab, ramucirimab, cetuximab, or panitumumab added for stage IV (181–186).

Regulation of gene expression by different types of non-coding RNAs such as miRNAs and lncRNAs are involved in acquisition of drug resistance characteristics after treatment (187). Most important dysregulated lncRNAs are summarized in Table 3. For instance, the characteristic acquisition of 5-FU resistance in CRC has been related with a plethora of lncRNAs miss-expression. In the case of UCA-1, it plays an important role in 5-FU chemoresponse by exerting a sponge activity to miR-204-5p, thus, indirectly increases CREB1 which have been related with poor OS (172). Another LncRNA implicated in the development of 5-FU resistance is GIHCG, since its overexpression is found in both CRC tissues and cell lines and is related to invasion, migration, and chemoresistant properties (188). There is also evidence that downregulation of PVT1, MALAT1, and PCAT-1 sensitizes CRC cells to 5-FU treatment, inducing early and late apoptosis by regulation of MDR genes (193, 194, 196). On the other hand, downregulation of snaR and SLC25A25-AS1 promotes chemoresistance in CRC (198, 199).

**Table 3:** Long non-coding RNAs and their physiological function in colorectal cancer drug resistance.

**LncRNA**	**Function**	**References**
GIHCG	Potential target in 5-FU and Oxaliplatin resistance mechanisms.	(188)
MIR100HG	Coordinately MIR100HG, miR-100 and miR-125b overexpression drives Cetuximab resistance by targeting five negative regulators of Wnt signaling which have a potential clinical relevant interaction with EGFR.	(189)
UCA1	UCA1 can decrease the sensitivity of CRC cells to 5-FU by sponging miR-204-5p resulting in attenuating apoptosis. Moreover, UCA1 expression levels are increased in Cetuximab resistant cells and can be transferred to sensitive cells through exosomes increasing resistant cells number.	(172, 190)
LINC00152	LIN00152 confers Oxa and 5-FU chemoresistance by sponging miR-193a-3p by ERBB4 modulation and then inducing the activation of AKT signaling pathway that mediates cell survival and chemoresistance. miR-193a-3p also targets NOTCH1 regulating CRC growth, metastasis, stemness, and chemoresistance.	(191, 192)
HOTAIR	HOTAIR could regulate the progression and Cisplatin and Paclitaxel chemoresistance enhancements in CRC by targeting miR-203a-3p and the activity of Wnt/β-catenin signaling pathway.	(116)
PCAT-1	PCAT-1 regulates the invasiveness and 5-FU resistance in CRC cells and that PCAT-1 may promote CRC cell invasion by modulating the expression of c-Myc.	(193)
PVT1	PVT1 is associated with 5-FU resistance in human CRC tissues and cells by inhibiting apoptosis and upregulating the expression of MRP1, P-gp, mTOR, and Bcl-2	(194)
XIST	XIST promotes Doxorubicin resistance through sponging miR-124 which targets SGK1 increasing cell survival, loss of control in cell cycle, inhibiting apoptosis, and increasing chemoresistance.	(195)
MALAT1	Overexpression of MALAT1 enhances chemoresistance in 5-FU resistant cells through potentiation of multidrug resistant genes such as MDR1, MRP1, BCRP, and ABC. Moreover, modulates EZH2 pathway in Oxa resistance	(196, 197)
H19	H19 mediated Methotrexate resistance via activating Wnt/β-catenin signaling, which help to develop H19 as a promising therapeutic target for MTX resistant CRC. Besides, CAFs promote stemness and Oxa chemoresistance in CRC by transferring exosomal H19 to CRC sensitive cells through sponging miR-141.	(20, 134)
SLC25A25-AS1	SLC25A25-AS1 has a pivotal role in CRC cells promoting chemo sensitivity to 5-FU and DOX via Erk and p38 pathway modulation. Hence, SLC25A25-AS1 was determined to play a tumor suppressive role in CRC.	(198)
snaR	snaR has a negative regulator role in responsible of the development of 5-FU resistance through cell growth of CRC cells. Nonetheless, snaR detailed roles have not yet been established.	(199)
ENST00000547547	ENST00000547547 reduced the chemoresistance of 5-FU via competitive sponging to miR-31 which targets ABCB9 involved in chemotherapy induced apoptosis. This suggests that lncRNA ENST00000547547 may be a positive prognostic factor for 5-FU-based chemotherapy.	(200)
TUG1	TUG1 mediates MTX resistance in colorectal cancer via sponging miR-186 that targets CPEB2 increasing its protein levels that play an important role in tumorigenesis and chemoresistance.	(201)
PVT1	PVT1 is a significant regulator in tumorigenesis and cisplatin resistance of CRC by inhibiting apoptotic pathways in CRC and may serve as a promising target for CRC therapy.	(202)
MEG3	MEG3 promotes chemosensitivity to Oxa by inducing cytotoxicity in CRC cells promoting apoptosis. In addition, MEG3 sponges miR-141 that targets PDCD4.	(203, 204)

*5-FU, 5-fluorouracil, Oxa, oxaliplatin. CAFs, cancer associated fibroblasts, DOX, doxorubicin*.

Certain aspects of chemoresistance have been related with lncRNAs regulated by miRNAs, for instance, ENST00000547547 promotes sensitivity to 5-FU in CRC cells by competitive arresting miR-31/ABCB9 (200) and LINC00152/miR-139-5p/NOTCH1 axis increases chemoresistance by suppressing apoptosis (191).

In the case of oxaliplatin CRC treatment, several lncRNAs such as GIHCG (172), LIN00152 (192), MALAT1 (197), H19 (20), and MEG3 (203, 204) promote apoptosis by inducing cytotoxicity by different mechanisms, mainly by axis with miRNAs targeting important genes in cell death behavior. Nevertheless, cisplatin CRC resistance is mainly mediated by HOTAIR and PVT1 through inhibition of apoptotic pathways, modulation of expression levels of miR-203a-3p and the activity of Wnt/β-catenin signaling pathway, respectively (116, 202).

Interestingly, H19 also exert drug resistance modulation in Methotrexate treatment via Wnt/β-catenin signaling pathway (134). Regarding to TUG1, the resistance is given by CPBE2 gene modulation after arresting of miR-186 (201). Finally, Doxorubicin resistance is manly influenced by the XIST/miR-124/SGK1 axis which promotes chemoresistance in CRC cells (195).

Evaluating lncRNAs expression profiles is very important since it can be used to identify novel biomarkers for CRC resistance and use them as a therapeutically potential targets based on their biological behavior, improving in this way, the efficacy of chemotherapy in CRC patients.

## Clinical Relevance on lNCRNA in Autophagy and Drug Resistance in Colorectal Cancer

Clinical implications of lncRNAs in CRC are relevant as there is evidence of its participation and correlation with staging and survival. In this regard, GAS5 down-regulation is common in CRC tissues being associated with distant metastasis, tumor differentiation, tumor size and advanced TNM staging (100), low histological grade (102), later tumor-node-metastasis stage and less OS (103).

Clinical relevance of H19 has been related with poor recurrent free survival (RFS) (9) tumor differentiation and advanced TNM stage, and is an independent predictor for OS and DFS. Moreover, previous studies using HOTAIR have determined that its overexpression is related to lymph node and, tumor node metastasis, distant metastases, Duke's staging, histological types, degree of differentiation (113) and poor clinical prognosis (114). Some studies show that it is upregulated in right CRCs biopsies (117). In addition, high levels of HOTAIR in tumors and blood are associated with higher mortality of patients (118).

MALAT1, patients have shown worse prognosis in tumors that appearance overexpression of this lncRNA in human primary CRC (145). In addition, MALAT1 have being related with lymph node metastasis in CRC patients (136).

Regarding to autophagy and chemoresistance in CRC, HAGLROS, a lncRNA related to autophagy, is correlated with shorter survival time (153). KCNQ1OT1, has also prove that induce protective autophagy and chemoresistance and its high expression is associated with poor OS of colon cancer patients, suggesting that patients with overexpression of KCNQ1OT1 might be resistant to chemotherapy treatments (173). Finally, H19 has been correlated with patient OS suggesting being a potential biomarker for predicting 5-FU resistance that could be modulated by H19/miR-194-5p axis (157).

## Concluding Remarks

Recently lncRNAs analysis is important for cancer detection, progression, diagnosis, therapy response, and prognostic values. With increasing development of quantitative detection techniques, lncRNAs derived from patients' non-invasive samples (i.e., blood, stools, and urine) has become into a novel approach in precision oncology.

Tumorspecific GAS5, HOTAIR, H19, and MALAT are novels CRC related lncRNAs detected in patients. Nonetheless, the effect and mechanism of lncRNAs in cancer autophagy and chemoresistance have not been extensively characterized.

Chemoresistance and autophagy are top issues for cancer treatment and lncRNAs play a pivotal role in resistance acquisition for several drugs. LncRNAs such as HAGLROS, KCNQ1OT1, and H19 are examples of lncRNA related to chemoresistance leaded by autophagy. Nevertheless, identifying the network interactions of lncRNAs can provide an insight in their mechanisms of action, adding clinical significance and hence, improve detection, diagnosis, and treatment.

## Author Contributions

MB, MA-M, EL-V, MA-F, and RR-P conceived and designed the content of this review and wrote the paper. ES-B and CL-C contributed to the final version of the manuscript.

### Conflict of Interest

The authors declare that the research was conducted in the absence of any commercial or financial relationships that could be construed as a potential conflict of interest. The reviewer NJ-H declared a past co-authorship with one of the authors, CL-C, to the handling editor.
